# Functional Connectivity of the Cognitive Cerebellum

**DOI:** 10.3389/fnsys.2021.642225

**Published:** 2021-04-08

**Authors:** Christophe Habas

**Affiliations:** Service de NeuroImagerie, Centre Hospitalier National d’Ophtalmologie des 15-20, Paris, France

**Keywords:** neocerebellum, crus 1–2, cognition, functional connectivity, resting-state, intrinsic networks

## Abstract

Anatomical tracing, human clinical data, and stimulation functional imaging have firmly established the major role of the (neo-)cerebellum in cognition and emotion. Telencephalization characterized by the great expansion of associative cortices, especially the prefrontal one, has been associated with parallel expansion of the neocerebellar cortex, especially the lobule VII, and by an increased number of interconnections between these two cortical structures. These anatomical modifications underlie the implication of the neocerebellum in cognitive control of complex motor and non-motor tasks. In humans, resting state functional connectivity has been used to determine a thorough anatomo-functional parcellation of the neocerebellum. This technique has identified central networks involving the neocerebellum and subserving its cognitive function. Neocerebellum participates in all intrinsic connected networks such as central executive, default mode, salience, dorsal and ventral attentional, and language-dedicated networks. The central executive network constitutes the main circuit represented within the neocerebellar cortex. Cerebellar zones devoted to these intrinsic networks appear multiple, interdigitated, and spatially ordered in three gradients. Such complex neocerebellar organization enables the neocerebellum to monitor and synchronize the main networks involved in cognition and emotion, likely by computing internal models.

## Introduction

The cerebellum has been classically involved in sensorimotor planning, execution, control, automation, and learning. However, in the last 30 years, a growing number of studies has broadened its role to cognitive and emotional processing. Anatomical tracing in monkeys showing cerebellum interconnections between neocerebellum and associative brain areas organized in closed loops (Schmahmann and Pandya, 1997; Strick et al., 2009) in agreement with human tractograms reviewed in Habas and Manto (2018), human clinical studies leading to the description of a “cerebellar cognitive affective syndrome” (Schmahmann and Sherman, 1998), and activation functional MRI (Stoodley et al., 2012; Stoodley and Schmahmann, 2018) have firmly supported the enlarged functional implication of the cerebellum in cognition. Moreover, from a phylogenetical standpoint, increased neocerebellar (posterior lobule) volume (MacLeod et al., 2003) and folding (Sereno et al., 2020), as well as an increased number of associative, mainly prefrontal, cerebello-cortical connections have been observed during the macroevolution history from great apes to humans (Ramnani et al., 2006; Balsters et al., 2010; Smaers, 2014). In other words, the neocerebellar expansion (lobules VII–VIII, especially crus 1–2) is parallel to the associative, mainly prefrontal, expansion in hominoids and humans.

In humans, two complementary imaging methods have been applied to delineate the cerebellar networks subserving cognitive functions. As mentioned above, diffusion imaging coupled with tractography strove to identify neocerebellar afferents and efferents connecting the cerebellum with associative cortices, thalamus, and striatum. The second method, on which we will exclusively focus, consists in determining the brain “resting-state” static and dynamic functional connectivity (rssFC). These methods allowed identifying the associative cortices functionally connected with the neocerebellum, and the whole network the neocerebellum takes part in.

## Functional Connectivity Methods

“Resting-state” static and dynamic functional connectivity detects temporal correlations between spontaneous BOLD signal fluctuations in a specific frequency domain (0.01–0.1 Hz) across brain areas belonging to specific—genetically prewired—networks during the brain “resting state.” Several algorithms (Bastos and Schoffelen, 2016) have been utilized to determine cerebellar rssFC, such as correlational, independent component, amplitude of low-frequency fluctuations, and regional homogeneity analyses. The most widely used ones are the seed-based correlational analysis and independent component analysis (ICA). The first method computes the r Pearson correlation coefficient between the BOLD time-series of a region of interest (ROI) and the time-series of the rest of the brain. It generates a specific temporal correlational map between the ROI and the functional interconnected brain areas. The second method consists of an exploratory multivariate data-driven approach. ICA decomposes the MRI dataset into statistically independent spatial maps, part of which can represent distinct large-scale networks whose neural nodes exhibit synchronized activity. The other part corresponds to different kinds of noise such as head or eye movements, breathing, heart rate, or spinal fluid pulsation. rssFC studies have identified the associative brain areas specifically connected with the neocerebellum, using seed-based method, and allowed to group these areas in functional networks, called intrinsically connected networks (ICNs), using ICA. However, these methods assume a stationary resting-state brain activity across the whole MRI exam and, thus, fail to describe the temporal dynamics of network recruitment. Dynamic functional connectivity methods have been developed to overcome this important limitation (Hutchison et al., 2013) such as the sliding window, time-frequency, paradigm-/parameter-free mapping, coactivation patterns (CAPs), or innovation-driven coactivation pattern (iCAP) methods (Preti et al., 2017). Put in a nutshell, the former technique relies on the segmentation of the BOLD time series in intervals of equal duration (usually around 30 s). Functional connectivity is then calculated for each interval separately, highlighting the temporal evolution of within- and between-network reconfiguration. Conversely, the CAP method associated with K-mean clustering consists in a point process analysis tracking brief (around 5–10 s) recurring coactivation or co-deactivation patterns by computing the rate of BOLD peaks or trough co-occurrence between an ROI and the rest of the brain voxels. In addition, the iCAP method specifically deals with transient encoding, in the BOLD fluctuations, onsets of network (de-)activations (Karahanoğlu and Van De Ville, 2015). All these methods permit to capture time-varying states characterized by synchronized networks and to quantify their duration (dwell time), the frequency of their occurrence, and the frequency of state-to-state transitions. Dynamic functional connectivity studies revealed that resting-state brain activity is a highly non-stationary process characterized by dynamic within- and across-network reconfiguration into recurring, sometimes overlapping, patterns (CAPs) and correlated with specific phases of the spontaneous low-frequency BOLD signal (Gutierrez-Barragan et al., 2019).

## Functional Connectivity and Graph Analysis

Graph analysis can be applied to functional connectivity in order to decipher network topological organization (Bullmore and Sporns, 2009). Brain circuits are regarded as graphs composed of a set of nodes (brain areas) interconnected by edges (functional and/or structural links). An edge between region A and region B is said to be “oriented or directed” if A exerts a causal effect on B (effective connectivity), as measured, for instance, by dynamic causal modeling or Granger causality. Such edges can also be weighted, for example, by the internode correlation coefficient. Several metrics have been defined to thoroughly describe the complex architecture of functional brain networks, such as connection or adjacency matrix, node connectivity degree, node connection strength, internodal path length, shortest path length, etc. (Bullmore and Sporns, 2009; Sporns, 2018). Networks encompass modules interconnected by specific nodes, called provincial hubs, and networks are bridged by connector hubs. Modules are implicated in local specialized information processing, whereas connector hubs contribute to information transferring and integration. Most networks display a specific architecture, called small-world architecture, which is intermediate between random (short path length between nodes) and regular organization (high clustering among nodes). Such small word architecture optimizes regional information processing and distributed integration. Small-world organization has been demonstrated in resting-state networks (Achard et al., 2006; van den Heuvel et al., 2008), and its graph properties varied in relation with the frequency of the BOLD fluctuations (Thompson and Fransson, 2015). Therefore, resting-state networks also undergo dynamic topological reconfiguration.

## Functional Connectivity Physiological Basis

The physiological mechanism underlying the endogenous hemodynamic low-frequency fluctuations remains a matter of debate. RssFC in the gray matter would derive from a region-specific complex combination of Fox and Raichle (2007): 1. (inter-) neuronal and astrocytic sources, such as spiking, quantal exocytosis, up–down neuronal states, energetic metabolism, extracellular sodium/potassium regulation (Krishnan et al., 2018), neuromodulation (Cole et al., 2013), microstates (Custo et al., 2017), topological network constraints (Deco and Corbetta, 2011), vasculature and extracerebral blood flow source (Tong et al., 2019), and behavioral sources (Lu et al., 2019). rssFC partly reflects the structural connectivity (SC) (Greicius et al., 2009) and can evolve with learning (epigenesis) in an age-dependent manner (Edde et al., 2020). Moreover, the cortical nodes of the resting-state networks display specific electroencephalographic power variation of infra-slow-to-gamma rhythms (Mantini et al., 2007; Grooms et al., 2017). In particular, there exists a strong correlation between infra-slow scalp potentials and the spontaneous BOLD signal (Hiltunen et al., 2014). Finally, biophysical models showed that the networks composed of coupled gamma oscillators linked by long-range structural connections with delay transmission yielded the emergence of endogenous low-frequency neural activity fluctuations (Cabral et al., 2017). In conclusion, rssFC is an emergent functional pattern of the brain activity, which is spatially and temporally multiscale organized from the cell to the network, and modulated by experience (training).

## Region of Interest-Based Associative Cerebello-Cortical “Resting-State” Static and Dynamic Functional Connectivity

fMRI studies (Stoodley et al., 2012; Stoodley and Schmahmann, 2018) using task-based protocols clearly delineated different sensorimotor territories such as sensorimotor (anterior lobe: lobules II–VI and VIIIB), oculomotor (vermis of lobules VI–VII, and lobules IX–X), vestibular (lobules IX–X), visual (lobule VI vermal), and auditory (lobules V–VI and left crus 1) zones. Cognitive and emotional regions have also been described, especially in lobule VII (Stoodley et al., 2012; Stoodley and Schmahmann, 2018). Using rssFC between the cerebellum and prefrontal cortex, Krienen and Buckner (2009) have found functional coherence between crus 2-lobule VIIB and the dorsolateral prefrontal cortex, crus 1-lobule IX and the medial prefrontal cortex, and VI/crus 1 border-crus 1-VIIB/VIIIA border and the anterior prefrontal cortex. O’Reilly et al. (2010) and Sang et al. (2012) have also found functional links between lobule VIIA paravermal-crus 2, posterior parietal and cingulate cortices as well as precuneus, lobule VIIA paravermal-IX and the prefrontal cortex, lobule VIII and the visual MT area, lobules VIII–IX and hippocampus and amygdala, and crus 1–2–vermal VIIIb–lobule IX and caudate nucleus. Regarding the vermis, rssFC has been identified between crus 2 and the cuneus, lobule VIIB and anterior thalamus–precuneus–posterior cingulate cortex, lobule VIIIA and superior frontal gyrus, and lobule IX and superior frontal and median temporal gyri (Bernard et al., 2012). All these studies have shown strong and lateralized functional coherence between crus 1–2 and contralateral prefrontal cortex. There exists a homotopic relation between associative cortical surface and their cerebellar representation with an over-representation within the cerebellar cortex of associative brain areas (Buckner et al., 2011).

The ROI-based rssFC demonstrates widespread interconnections between the neocerebellum (lobule VII and VIII) and prefrontal, parietal, cingulate, temporal, and occipital cortices. Such functional connections might rely on cortico-pontine afferents and/or cerebello-thalamo-cortical efferents in agreement with anatomical tracing in animal (Schmahmann and Pandya, 1997; Strick et al., 2009) and human tractography (Habas and Manto, 2018).

## Region of Interest-Based Dentato-Cortical “Resting-State” Static and Dynamic Functional Connectivity

Moreover, the human dentate nuclei, the main cerebellar output system to the brain and brainstem, exhibits rssFC with occipital (BA 19), parietal (BA 40), insular (BA 13), cingulate (BA 24), and prefrontal (BA6-8-9-32-46) cortices, the left dentate nucleus displaying more widespread efferents than the right one (Allen et al., 2005). The neocerebellar cortex, especially the prominent lobule VII, and the dentate nuclei constitute a supramodal zone interconnected with the major associative brain regions (O’Reilly et al., 2010) and, to a lesser extent, to affective and associative subcortical nuclei such as the amygdala and striatum. Finally, the interlobular rssFC could subserve a cross-network coordination within the cerebellum; for instance, crus 1–2 are correlated with lobule IX. This could functionally bridge executive network (EN) and default-mode network (DMN) (Bernard et al., 2012). Of interest, topological properties of the intracerebellar rssFC, such as small-world organization, depend upon intelligence coefficient and gender (especially in lobules VI–crus 1 on the left side and vermal VIII) (Pezoulas et al., 2018).

In conclusion, the cerebellum can influence, through dentate-thalamo-cortical projections, all the associative cortices.

## Independent Component Analysis-Based Associative Cerebello-Cortical Closed Loops

**FIGURE 1:**
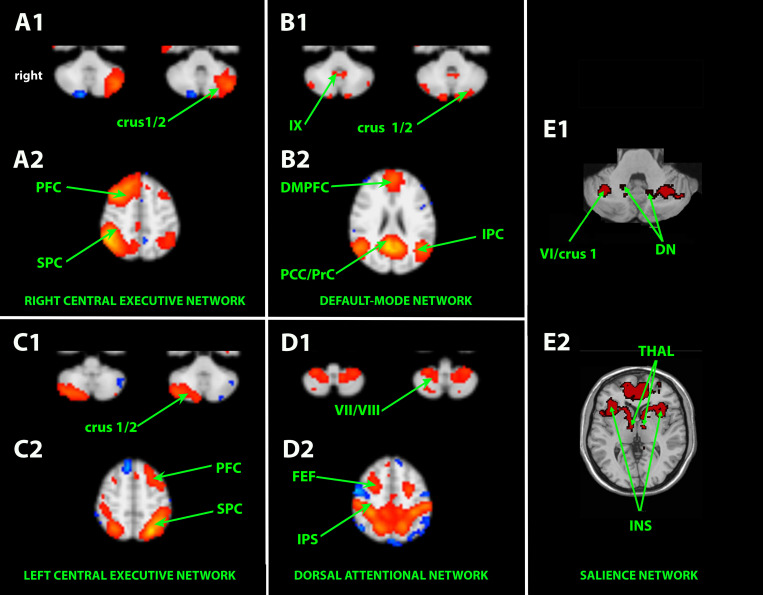
Independent component analysis (ICA)-based resting-state associative cortico-cerebello-cortical networks. A1–B1–C1–D1–E1: axial slices passing through the neocerebellum. A2–B2–C2–D2–E2: axial slices passing through the brain. DMPFC, dorsomedial prefrontal cortex; DN, dentate nucleus; FEF, frontal eye field; INS, insula; IPC, inferior parietal cortex; PCC/PrC, posterior cingulate cortex/precuneus; PFC, prefrontal cortex; SPC, superior parietal cortex; Thal, thalamus. Cerebellar lobules are numbered with Latin numerals. Crus 1/2 corresponds to the hemisphere of lobule VIIA.

The abovementioned cerebellar areas and functionally associated cortical areas take part in parallel cerebro-ponto/reticulo-cerebello-thalamo-cortical loops. More precisely, these ICNs encompass (Habas et al., 2009; Krienen and Buckner, 2009; Brissenden et al., 2016) (Figure 1):

- the right and left frontoparietal EN passing through crus 1–2 (working memory, adaptive control, and task switching),
- the DMN passing through crus 1–2 and lobule IX (mind wandering, episodic memory, agentivity, navigation, self-reflection, and consciousness),
- the limbic salience network passing through lobules VI–VIIb/crus 1–2 (interoception, autonomic regulation, emotional processing, and bottom–up attention),
- the frontoparietal dorsal attentional network (DAN) passing through lobules VIIB–VIIIA (top–down attention and visual working memory),
- the language-dedicated network passing through the cerebellum (especially right crus 1–2) (Tomasi and Volkow, 2012).

Using another method (fuzzy-c means clustering algorithm), Lee et al. (2012) also reported during the resting state a ventral attentional network (VAN) previously described by Corbetta et al. (2000). VAN encompasses mainly the inferior and middle prefrontal, temporoparietal junctional, anterior insula, inferior parietal on the right side, and is involved in the bottom–up reorientation of attention. VAN passes bilaterally through parts of lobules VI and VIIIA and, to a lesser extent, lobules crus 1 and VIIIB (Guell et al., 2018; Guell and Schmahmann, 2020). This circuit can switch DAN activity to a novel object of interest. It is noteworthy that several nodes of VAN, such as anterior insula, also belong to the limbic salience network.

In conclusion, the neocerebellum can influence all the associative resting-state networks.

## Gradient Organization

It has been shown (Guell et al., 2018; Guell and Schmahmann, 2020) that EN, DMN, and DAN are represented three times in each hemisphere of the neocerebellar cortex (lobules VII–VIII–IX–X), and that these representations are included in functional gradients from attentional (DAN) and task-positive executive (CEN) processing to task-negative default-mode processing (DMN). These three gradient-based representations are found in lobules VI–crus 1, lobules VIIB–crus 2, and lobules IX–X. The rostro-caudal direction of the first representation and the opposite direction of the second representation imply that the crus 1–2 intersection encompasses partial overlapping of the first and second DMN representations. These anatomical gradients mirror hierarchical cognitive control of the prefrontal and parietal cortices (D’Mello et al., 2020). Furthermore, this anatomo-functional gradient organization may reflect the phylogenetical coupling between telencephalization and neocerebellar development with progressive complexification of motor abilities requiring more executive control (attention, anticipation, and regulation) and behavioral integration (emotion-related behaviors) until the cerebellum could also monitor non-motor tasks. The differential role of these multiple representations and whether adjacent contiguous representations, as in the DMN case, would participate in a coordinated computation through, for instance, intracerebellar interconnections, remains to be determined.

## Emotional Cerebellum

It is worth noting that the “emotional cerebellum” belongs to SN and DMN, and includes, in particular, the vermis of lobule VII, in accord with the “constructivist” or “scaffolding” hypothesis, claiming that no specific network is dedicated, at least, to emotion such as lateral and medial pain matrix (Iannetti and Mouraux, 2010). Emotions rest on the transient collaboration of distinct intrinsic networks with specific hubs such as insula and anterior cingulate cortex (Menon and Uddin, 2010) and subserving the multidimensional (autonomic, affective, cognitive, mnesic, and motor) aspects of emotion.

## Striato-Cerebellar Interconnection

Of interest, several studies found structural and functional connectivity between the cerebellum and the limbic ventral striatum. For instance, Pelzer et al. (2013) found dentato-thalamo-striato-pallidal and sub-thalamo–pontine nuclei–cerebellar connections passing through crus 2/lobules VIII–IX, using probabilistic tractography. Functional coherence was recorded between lobule IX (DMN) and the ventral tegmental area (Murty et al., 2014), and between lobule VII–IX (EN, DMN, and SN) and nucleus accumbens (Cauda et al., 2011). In this vein, a serial reaction time task was accompanied by FC strengthening of a cerebello (crus 1 and dentate nucleus)-thalamo-lenticular nucleo-cortical network during explicit and implicit learning (Sami et al., 2014). Therefore, SC and FC tightly and directly interconnect the (neo-)cerebellum and basal ganglia, which explains why a reward signal can be detected in granule cells and climbing fibers (Wagner and Luo, 2020). Although the role of this latter signal requires further investigations, it has been speculated that the basal ganglia would send to the cerebellum a value estimation of the cerebellar forward model-based selection of cortical planned or executed mental actions (Caligiore et al., 2017). In other words, striatal reinforcement learning could not only modulate the cortical activity but also the cerebellar error-based supervised learning.

## Lobule VII “Resting-State” Static and Dynamic Functional Connectivity

Two main networks occupying the most voluminous neocerebellar lobule (VII) are represented by EN and DMN, with an EN predominance. The DMN-related cerebellar zone within crus 1–2 is surrounded by the EN-related cerebellar zone. It has been demonstrated that there exists a greater individual variability in the spatial organization of ICNs within the neocerebellum than in the corresponding cortex, despite a group-level identical spatial pattern, and that the resting state cerebellar fluctuations of EN and DMN lag behind the cortex ones by hundreds of milliseconds (Marek et al., 2018). Marek et al. (2018) hypothesized that the cerebral cortex would transmit information to the neocerebellum using infra-slow activity conveyed by cortico-ponto-cerebellar afferents, and the cerebellum would respond by sending a signal back to the cortex through the cerebello-thalamo-cortical efferents using delta rhythm (0.5–4 Hz). It is noteworthy that delta rhythms are involved in learning-dependent timing (Dalal et al., 2013), and in the latter assumption, part of the endogenous fluctuations could subserve information processing.

Intermittent theta burst magnetic stimulation applied to the neocerebellum induced DMN and EN reconfiguration in terms of functional connectivity and frequency of their associated electroencephalogram signal (Halko et al., 2014; Farzan et al., 2016). The stimulation of vermian lobule VII influenced the DAN with enhanced power in beta/gamma oscillations, whereas the stimulation of the hemisphere of lobule VII modulated the activity of DMN with diminished frontal theta activity. The absence of EN implication could be ascribed to the prominent recruitment of DMN during the resting state. This study illustrated that neocerebellum can differentially alter electrophysiological activity of networks.

Dynamic rssFC, studying time-varying rssFC, coupled with SC reveals the highest rssFC/SC similarity in the posterior lobe compared with the anterior one and a low rssFC variability (Fernandez-Iriondo et al., 2020). These last findings might explain the specific and constant recruitment of DMN and EN during mind wandering (Fox et al., 2016), and the high and temporally stable constraints exerted by SC onto the rssFC of the cognitive circuits. Moreover, the CAPs method was applied to the resting-state BOLD fluctuations (Liu and Duyn, 2013). When the left intraparietal sulcus belonging to DAN was seeded, several CAPs were found in distinct non-overlapping neocerebellar regions showing activation or deactivation. Thus, different transient states of a specific circuit can recruit distinct cerebellar subregions.

## Functional Considerations and Synthesis

The polymodal neocerebellum (lobules VII, VIII, and IX) is massively interconnected with associative cortices, as well as with the striatum and amygdala, and it partakes in all associative resting-state circuits with an overrepresentation of EN. Each circuit contributes to a triple functional gradient-based representation within the cerebellar cortex. These resting-state circuits are also characterized by a specific BOLD and electrophysiological signature. The slow BOLD fluctuations, at least for CEN, lag behind the cortical oscillations. This resting-state functional architecture can also be modulated by experience and individual mental abilities. For instance, enhanced functional coherence between crus 1–2 and the right CEN is positively correlated with task goal maintaining (Reineberg et al., 2015). However, functional connectivity analyses *per se* cannot specify which precise functional action is exerted by such networks. It is assumed that “[…] resting state networks represent a finite set of spatiotemporal basis function from which task-networks are then dynamically assembled and modulated during different behavioral states” (Mantini et al., 2007) even if intrinsic networks, especially DMN, can be actively recruited by mind wandering during the brain resting state. The associative function of the human neocerebellum can only be inferred from task-based fMRI paradigms and from clinical studies. Task-based fMRI meta-analysis has shown involvement of the neocerebellum, including lobules VI, VII, and VIII, in executive, linguistic, and emotional functions (Stoodley et al., 2012). In addition, cerebellar stroke patients exhibited cognitive deficits due to posterior lobe, mainly lobules VII–VIII, and dentate nucleus lesions (Stoodley et al., 2016). Such deficits corresponded to components of the Schmahmann’s cognitive and affective syndrome (Schmahmann and Sherman, 1998). Furthermore, from a computational standpoint, because of the structural and histological homogeneity of the cerebellum organized in microcomplexes, it is postulated that motor and associative cerebellum may accomplish the same algorithmic function. Substantial data support the view that the cerebellum may elaborate internal models and especially forward models (Wolpert et al., 1998; Sokolov et al., 2017). Such models would allow prediction of the consequences of intended mental activity during movement (sensory consequence of planned or executed motor action) (Ito, 2005) or cognition (Ito, 2008), and, consequently, would control and optimize the accuracy of the current performance, particularly during supervised learning. The cerebello-cortical closed loops would likely help coordinate or (de-)synchronize cortical areas through the thalamus reviewed in Habas et al. (2019) and to sequence their activity (Molinari et al., 2008). In other words, the cerebellum would act as a general modulator, or “universal transform” (Sereno and Ivry, 2019), generating internal models (functional unicity of microcomplexes) for all motor and associative/emotional domains (functional heterogeneity due to its wide interconnections with the cerebral cortex). Finally, it is noteworthy that task-free and task-based functional parcellations of the cerebellum can exhibit some small regional differences (King et al., 2019): for example, several tasks such as hand movement, working memory and language activation recruit bilateral homologous cerebellar zones, although these zones belong to lateralized resting-state networks. Moreover, if task-free intrinsic connectivity can predict task-evoked activations, small differences can be noted between the former one and the task-state functional connectivity (Cole et al., 2021). Idiosyncratic mental strategies to solve the current tasks may explain these differences observed in functional connectivity.

## Conclusion

“Resting-state” static and dynamic functional connectivity sheds light on the genetically prewired and epigenetically tuned resting-state networks underlying the cognitive function of the neocerebellum for motor and non-motor tasks. RssFC demonstrated in humans that the major part of the cerebellum (lobules VII–VIII and dentate nucleus) is functionally interconnected with non-motor associative cortices and constitutes a major relay of all associative resting-state networks. These cerebello-cortical functional interconnections partly reflect the underlying structural hardware. Further studies are required to determine whether this cerebello-cortical coherence would also reflect information processing/transferring between the cerebellum and its targets during the resting state. However, this “functional tracing” method does not furnish any explanation concerning the exact functional or algorithmic role of the neocerebellum in the cognitive domain. It can only suggest that the cerebellar computation based on supervised and predictive control through internal models—the current prevalent hypothesis about the cerebellar function—is the same for networks in charge of movements and networks in charge of executive and emotional processing. Finally, the advances in our understanding of the organization of ICNs would lead to a better understanding of the pathogenesis and therapy of major disorders of the brain such as Parkinson’s disease (Mueller et al., 2019) or genuine disorders of the cerebellar circuitry itself (Iang et al., 2019). The understanding of node shaping and synchronizing activities of the cortical areas (Habas et al., 2019) will benefit from refinements in the techniques currently applied, such as neurostimulation.

## Author Contributions

The author confirms being the sole contributor of this work and has approved it for publication.

## Conflict of Interest

The author declares that the research was conducted in the absence of any commercial or financial relationships that could be construed as a potential conflict of interest.
